# The immune status of migrant populations in Europe and implications for vaccine-preventable disease control: a systematic review and meta-analysis

**DOI:** 10.1093/jtm/taae033

**Published:** 2024-02-29

**Authors:** Zeinab Cherri, Karen Lau, Laura B Nellums, Jan Himmels, Anna Deal, Emma McGuire, Sandra Mounier-Jack, Marie Norredam, Alison Crawshaw, Jessica Carter, Farah Seedat, Nuria Sanchez Clemente, Oumnia Bouaddi, Jon S Friedland, Michael Edelstein, Sally Hargreaves

**Affiliations:** The Migrant Health Research Group, Institute for Infection and Immunity, St Georges, University of London, London, UK; The Migrant Health Research Group, Institute for Infection and Immunity, St Georges, University of London, London, UK; Department of Global Health and Development, Faculty of Public Health and Policy, London School of Hygiene and Tropical Medicine, London, UK; Lancet Migration European Regional Hub; Faculty of Medicine and Heath Sciences, University of Nottingham, Nottingham, UK; The Migrant Health Research Group, Institute for Infection and Immunity, St Georges, University of London, London, UK; The Migrant Health Research Group, Institute for Infection and Immunity, St Georges, University of London, London, UK; Department of Global Health and Development, Faculty of Public Health and Policy, London School of Hygiene and Tropical Medicine, London, UK; The Migrant Health Research Group, Institute for Infection and Immunity, St Georges, University of London, London, UK; Department of Global Health and Development, Faculty of Public Health and Policy, London School of Hygiene and Tropical Medicine, London, UK; Danish Research Centre for Migration, Ethnicity and Health, Section of Health Services Research, Department of Public Health, University of Copenhagen, Denmark; Department of Infectious Diseases, Hvidovre Hospital, University of Copenhagen, Denmark; The Migrant Health Research Group, Institute for Infection and Immunity, St Georges, University of London, London, UK; The Migrant Health Research Group, Institute for Infection and Immunity, St Georges, University of London, London, UK; The Migrant Health Research Group, Institute for Infection and Immunity, St Georges, University of London, London, UK; The Migrant Health Research Group, Institute for Infection and Immunity, St Georges, University of London, London, UK; Lancet Migration European Regional Hub; International School of Public Health, Mohammed VI University of Sciences and Health, Casablanca, Morocco; Mohammed VI Center for Research and Innovation, Rabat, Morocco; Institute for Infection and Immunity, St Georges, University of London, London, UK; Independent Public Health Consultant, London, UK; The Migrant Health Research Group, Institute for Infection and Immunity, St Georges, University of London, London, UK; Lancet Migration European Regional Hub

**Keywords:** Vaccination, immunization, migrant, measles, mumps, rubella, diphtheria

## Abstract

**Background:**

Ensuring vaccination coverage reaches established herd immunity thresholds (HITs) is the cornerstone of any vaccination programme. Diverse migrant populations in European countries have been associated with cases of vaccine-preventable diseases (VPDs) and outbreaks, yet it is not clear to what extent they are an under-immunized group.

**Methods:**

We did a systematic review and meta-analysis to synthesize peer-reviewed published primary research reporting data on the immune status of migrants in EU/EEA countries, the UK and Switzerland, calculating their pooled immunity coverage for measles, mumps, rubella and diphtheria using random-effects models. We searched on Web of Science, Embase, Global Health and MEDLINE (1 January 2000 to 10 June 2022), with no language restrictions. The protocol is registered with PROSPERO (CRD42018103666).

**Findings:**

Of 1103 abstracts screened, 62 met eligibility criteria, of which 39 were included in the meta-analysis. The meta-analysis included 75 089 migrants, predominantly from outside Europe. Pooled immunity coverage among migrant populations was well below the recommended HIT for diphtheria (*n* = 7, 57.4% [95% confidence interval (CI): 43.1–71.7%] *I*^2^ = 99% vs HIT 83–86%), measles (*n* = 21, 83.7% [95% CI: 79.2–88.2] *I*^2^ = 99% vs HIT 93–95%) and mumps (*n* = 8, 67.1% [95% CI: 50.6–83.6] *I*^2^ = 99% vs HIT 88–93%) and midway for rubella (*n* = 29, 85.6% [95% CI: 83.1–88.1%] *I*^2^ = 99% vs HIT 83–94%), with high heterogeneity across studies.

**Interpretation:**

Migrants in Europe are an under-immunized group for a range of important VPDs, with this study reinforcing the importance of engaging children, adolescents and adults in ‘catch-up’ vaccination initiatives on arrival for vaccines, doses and boosters they may have missed in their home countries. Co-designing strategies to strengthen catch-up vaccination across the life course in under-immunized groups is an important next step if we are to meet European and global targets for VPD elimination and control and ensure vaccine equity.

## Introduction

Migration to and within Europe has steadily increased in recent years, involving a mix of both individuals born in the European Union (EU) or European Economic Area (EEA) but living outside of their EU country of birth as well as born outside of the EU/EEA. ^1^ Migrants (defined as ‘foreign born’) are a heterogeneous group with diverse health needs,^1–4^ and include refugees and asylum seekers who have been forcibly displaced due to conflict and persecution—as well as undocumented migrants and a growing number of labour migrants.^5–7^ Some migrant communities in Europe—particularly adolescent and adult migrants arriving from low-income and middle-income countries—are at high risk of under-immunization for routine vaccinations resulting from missed routine vaccines, doses and boosters as children in their home countries and their marginalization from health and vaccination systems in transit and host countries^8^; however, this has been poorly quantified to date. There are also known to be a range of factors driving under-immunization and hesitancy in migrant populations, including unique awareness and access factors that need to be better considered in policy and service delivery.^9^ This potentially places them at increased risk of being involved in outbreaks and morbidity and mortality from vaccine-preventable diseases (VPDs).^10–16^ The vulnerability of populations to VPDs has been exacerbated by the disruptive impact of the COVID-19 pandemic on routine immunization programmes.^17^ The European Centre for Disease Prevention and Control (ECDC) has highlighted the risk of measles importation and reintroduction in European countries, and this may be exacerbated post-COVID-19 pandemic with a decline in measles coverage rates.^18^ The WHO’s new Immunisation Agenda 2030 (IA2030),^19^ and subsequent WHO reports^20^^,^^21^ call on European countries to work towards achieving or sustaining the elimination of measles, rubella and polio and controlling hepatitis B infection, acknowledging that particular attention will need to be given to marginalized and vulnerable groups across the life course—including migrants and other under-immunized groups—and that vaccine service delivery strategies will need to be tailored at national and subnational levels. WHO’s recent European Immunisation Agenda 2030 ^22^ specifically calls for states to ensure all groups have equitable access to vaccine services and to identify and offer vaccination to all people who have missed vaccinations. However, migrants are rarely considered in vaccination programmes on arrival to European countries.^23^ Although national vaccination guidelines exist in most European countries, very few of these guidelines have a specific migrant focus, and in practice, there is a clear gap in their effective implementation particularly for adolescent and adult migrants.^24^

Comprehensive datasets are lacking for health planners and policymakers to fully understand levels of under-immunization and the burden of VPDs among migrant populations in the EU/EEA countries. Although WHO and ECDC publish country data on vaccination coverage by vaccine, these data are not disaggregated by migrant status. Heterogeneity among published studies, including populations and study design, also pose a challenge in understanding the current state of knowledge on the immune status of migrants. In the last decade, migrants have been associated with outbreaks of VPDs such as measles and diphtheria in Europe,^13^^,^^16^^,^^25^^,^^26^ including a large pan-European 2017–20 measles epidemic with the highest numbers of cases and deaths witnessed in decades.^14^^,^^27^ The ECDC has also reported a recent increase in diphtheria cases, mostly among asylum seekers, with 153 cases reported by eight European countries in 2022 among migrants, resulting in one death.^15^ A recent outbreak of diphtheria in asylum accommodation in the UK further reinforced the importance of engaging adolescents and adults in ‘catch-up’ vaccines on arrival for vaccines, doses and boosters they may have missed in their home countries as children.^16^^,^^28^

Ensuring that migrants, and other groups who may be at risk of under-immunization, are fully vaccinated in line with vaccination schedules in EU/EEA countries is vital to achieving population-level herd immunity to prevent disease, disability and mortality from VPDs. Vaccination increases the number of immune individuals in a population, acting as a barrier to disease transmission. Once a critical proportion of the population develops immunity, the herd immunity threshold (HIT) is reached.^29^ To achieve herd immunity for measles requires vaccine coverage rates as high as 93–95%, mumps 88–93%, rubella 83–94% and diphtheria 83–86%.^30–32^ Achieving HIT enables transmission to stop within the given population.^33^ Achieving HITs is, however, not sufficient, and maintaining vaccine coverage at or above the HIT is needed to eliminate or control VPDs (e.g. WHO has set targets of 95% coverage for the first dose of MMR for 5-year-old children).^30^ Many EU/EEA countries with long implemented vaccination plans show coverage short of these HITs that have further decreased since the COVID-19 pandemic and place under-immunized populations at greater risk. In Italy, e.g. timely coverage for the second dose of a measles containing vaccine (MCV2) was 85% in 2022, compared with 88% in 2019.^34^ Many conflict-affected or low-income countries where many migrants are coming from fail to meet 50% coverage.^12^ For example, MCV2 coverage in 2022 was 38% in Syria and 49% in Afghanistan.^34^

Achieving and maintaining HIT across population groups is a cornerstone of preventing VPD cases and outbreaks. However, the extent to which migrants represent an under-immunized population in the European context has not been formally assessed for key VPDs, hampered by poor data collection and weak surveillance systems. We therefore did a systematic review and meta-analysis to comprehensively identify and synthesize data on the immune status of migrants (defined as foreign born) in EU/EEA countries, the UK and Switzerland, calculating their pooled immunity coverage for measles, mumps, rubella and diphtheria.

## Methods

### Search strategy and inclusion/exclusion criteria

We carried out a systematic literature review and meta-analysis in line with Preferred Reporting Items for Systematic Reviews and Meta-Analyses (PRISMA) guidelines.^35^ The protocol was prospectively registered on PROSPERO (CRD42018103666). We searched Web of Science, Embase, Global Health and MEDLINE for peer-reviewed primary research reporting on immune status (e.g. vaccination history or laboratory confirmation) in migrant populations in the EU/EEA, the UK and Switzerland between 1 January 2000 and 10 June 2022, with no language restrictions. Following an iterative process of searching relevant systematic reviews and consulting with experts in the field, a Boolean search strategy was developed containing terms pertaining to migration, VPDs, vaccination and immunity (see Supplementary file for full search strategy). We searched for studies from all EU/EEA countries, the UK and Switzerland (Austria, Belgium, Bulgaria, Cyprus, Croatia, Czech Republic, Denmark, Estonia, Finland, France, Germany, Greece, Hungary, Iceland, Ireland, Italy, Latvia, Liechtenstein, Lithuania, Luxembourg, Malta, the Netherlands, Norway, Poland, Portugal, Romania, Slovakia, Slovenia, Spain, Sweden).

We defined a migrant as any foreign-born individual, born outside the country in which data were collected or reported. We included peer-reviewed citations reporting primary data from observational studies (e.g. cross-sectional, case–control or cohort studies). Comments, editorials, systematic reviews and letters were excluded. Non-English papers were included to be representative of migration and VPD research occurring across Europe. Studies were eligible for inclusion if they reported primary data on immune status or vaccination status for migrants to the EU/EEA, the UK and Switzerland (from any low- middle- or high-income country) disaggregated by country of birth or foreign-born status for the following infections: measles, mumps, rubella and diphtheria. All age groups and immune status indicators (laboratory-tested immune status/serology, self-reported vaccination status or registries/clinical records for vaccinations given) were included in the review.

For the meta-analyses, the inclusion criteria to enable pooled immunity coverage to be estimated were serology studies reporting primary data disaggregated by migrant status including the total migrant sample (*N*) and laboratory confirmation of vaccination status [percentage of number (*n*)]. For studies reporting on multiple VPDs, relevant data were included in each disease-specific meta-analysis. Exclusion criteria were citations in which data were not transparently reported for migrants or disaggregated by migrant (foreign-born) status or VPD. The primary outcome was immune status, including immunity indicators (e.g. seroprevalence, according to recognized cut-off criteria), which was used to estimate the pooled immunity coverage for migrant populations in the meta-analyses.

### Data screening, extraction and synthesis

We did title and abstract screening, full-text screening, data extraction and quality assessment of included studies, all of which was duplicated by an independent second reviewer, in line with PRISMA guidelines. All titles and abstracts were screened for their relevance and eligibility. Full-text screening was then carried out for all potentially eligible studies, and reasons for exclusion were recorded. Differences in screening decisions were discussed between the reviewers until consensus was reached. Data extraction tables were created and data from the included studies were extracted on the following: study design, location, population, sample size, migrant proportion of sample, sample demographics (including age group, gender and countries/areas of origin), reported vaccination status and immune status (e.g. measured antibody titres).

### Quality assessment

Quality assessment of the included studies was carried out independently by two reviewers using the Joanna Briggs Institute (JBI) critical appraisal tool^36^ for cross-sectional and cohort studies. A total of eight points could be allocated to each study, with scores of 6–8 considered high quality. Studies were not excluded based on quality score in order to increase transparency and report on all studies meeting the inclusion criteria. However, sensitivity analyses were carried out to examine how study quality influenced the results. Two reviewers carried out the quality assessments, with differences in scores discussed until consensus was reached. Where decisions could not be reached, two further reviewers arbitrated.

### Statistical analysis

Data were extracted on a priori forms and imported into and analysed using R (version 4.2.1). Where appropriate, the command *metaprop* was used for the meta-analysis of binomial data to calculate pooled immunity coverage and 95% confidence intervals for the separate VPDs examined.^37^ Heterogeneity was quantified using the *I^2^* statistic,^38^ and the range of immunity coverage across studies for each disease was reported. The higher the *I^2^* statistic, the greater the heterogeneity. Due to the expected heterogeneity between studies, we used random-effects models for the analyses.^39^ The random-effects model is preferred over the fixed-effects model because the random-effects model assumes that the true effect could vary from study to study due to the differences among studies and therefore allows for the possibility that studies in a meta-analysis have heterogeneous effects. Subgroup analyses were also carried out for each VPD included in the meta-analysis to examine immunity coverage by age group (e.g. adult vs children).

## Results

### Summary of included studies

**Figure 1:**
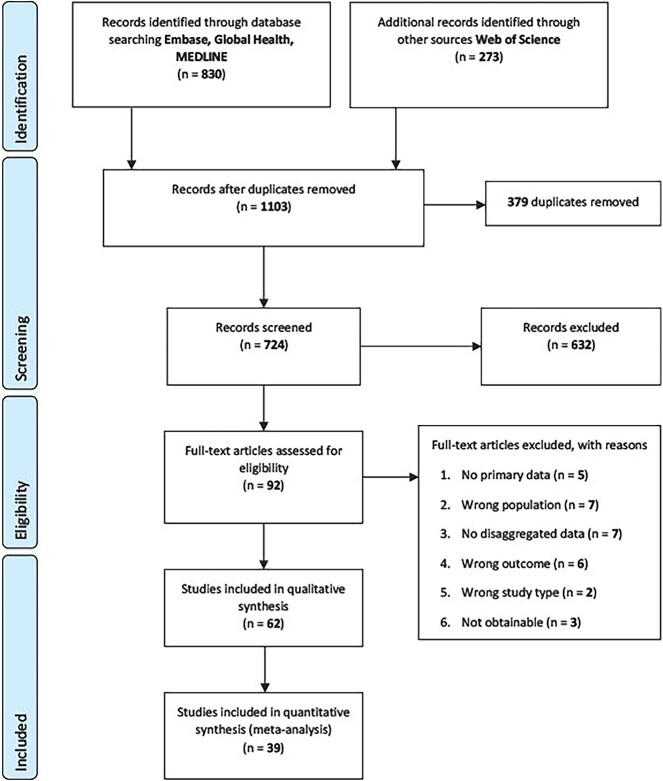
PRISMA diagram of included studies

We identified 1103 citations through the database searches. After removing 379 duplicates, 724 unique papers were included in the title and abstract screening, of which 632 were excluded. 92 full-text papers were screened and of these, 62 studies met the inclusion criteria and were included in the review (Figure 1). For the meta-analyses of serology/laboratory studies only, 39 studies involving 75 089 migrants were included.

The review included 62 studies from 14 countries (see Table S1): Austria (*n* = 3),^40–42^ Denmark (*n* = 4),^43–46^ France (*n* = 4),^13^^,^^47–49^ Germany (*n* = 15),^50–64^ Greece (*n* = 2),^65^^,^^66^ Ireland (*n* = 1),^67^ Italy (*n* = 9),^68–76^ Luxembourg (*n* = 1),^77^ the Netherlands (*n* = 1),^78^ Norway (*n* = 1),^79^ Spain (*n* = 13),^80–92^ Sweden (*n* = 2),^93^^,^^94^ Switzerland (*n* = 3),^95–97^ Switzerland & Germany (*n* = 1)^98^ and the UK (*n* = 2).^99^^,^^100^ Fifty-four studies were cross-sectional, 2 studies were cohorts and 6 reported on outbreaks.^13^^,^^55^^,^^61^^,^^62^^,^^83^^,^^98^ The majority of studies (39 of 62 studies) used serology to assess immune status, 2 used PCR and the other studies were a mix of self-reported vaccination status and data acquired through registries and clinical records.

The meta-analyses included 39 studies^42–44^^,^^49^^,^^50^^,^^53^^,^^54^^,^^59^^,^^63^^,^^64^^,^  ^66–70^^,^^73^^,^^74^^,^^76–82^^,^^84–97^^,^^99^ reporting disaggregated data on immune status using serology in migrants in 14 European countries: Austria (*n = 1*), Denmark (*n* = 2), France (*n = 1*), Germany (*n* = 6), Greece (*n* = 1), Ireland (*n* = 1), Italy (*n* = 6), Luxembourg (*n* = 1), The Netherlands (*n* = 1), Norway (*n* = 1), Spain (*n* = 12), Sweden (*n* = 2), Switzerland (*n* = 3) and the UK (*n* = 1). Twenty studies included in the meta-analysis reported on a single disease, and 19 reported on multiple diseases (Table S1): measles (*n* = 21), mumps (*n* = 8), rubella (*n* = 29) and diphtheria (*n = 7*). Studies were published between 2020 and 2022 (*n* = 4), 2011 and 2019 (*n* = 24) and 2000 and 2010 (*n* = 11). Twenty-six studies looked at sub-populations only, including pregnant women (*n* = 13),^50^^,^^67^^,^^79^^,^^82^^,^^86^^,^^88–93^^,^^96^^,^^99^ human immunodeficiency virus (HIV)-positive patients (*n* = 2),^49^^,^^85^ women with chronic hepatitis B (*n* = 1),^43^ and asylum seekers or refugees (*n* = 11).^44^^,^^50^^,^^53^^,^^54^^,^^63^^,^^64^^,^^70^^,^^76–78^^,^^94^ Six studies focused on children and/or adolescents, including four focusing specifically on internationally adopted children.^68^^,^^69^^,^^73^^,^^80^ Seventeen studies used adults as their target group, and 16 studies were made up of mixed-age cohorts. Twenty-one studies took place in primary care and antenatal clinics, 9 involved individuals housed in refugee camps or asylum centres, and 2 analysed migrant immune status on arrival to the host country.

We conducted quality assessment using the JBI critical appraisal tool^36^ for cross-sectional and cohort studies (see supplementary file Table S1). Only four studies could not be assessed because they included results from outbreak interventions. Only four studies had a minimum score of 3, while all other studies had a score of 5 and above, with scores of 6–8 considered high quality. Overall, the quality of included studies was medium to high (Table S1). Studies were not excluded on the basis of quality, and study quality was not found to significantly impact on the findings in sensitivity analyses.

The migrants’ countries or regions of origin were not always specified, but studies included migrants originating from all regions in the world, predominantly outside Europe (Asia, Africa, Eastern Mediterranean), but also internal EU/EEA migrants who were mainly refugees from Albania, Kosovo or other Balkan countries.^66^^,^^77^

### Measles immunity

Thirty-seven studies (21 serology and 16 vaccination history) reported on measles in migrant populations^13^^,^^40^^,^^43–46^^,^^48^^,^^50–63^^,^^65^^,^  ^66^^,^^68–70^^,^^73^^,^^77^^,^^78^^,^^80^^,^^84^^,^^85^^,^^87^^,^^94^^,^^95^^,^^97^^,^^100^ (Table S1), predominantly among migrant children and in most cases reporting low levels of protective immunity. In Germany, one population-based study found that 77.5% [95% confidence interval (CI) 72.5–81.9] of migrant children and adolescents were considered to be immune to measles^59^ and another involving 23 647 asylum seekers reported serological immunity at 79.9% (CI 79.4–80.4%) with significant variation by country of origin.^63^ A 2019 serology study in Sweden with 1909 newly arrived immigrants reported 78% (95% CI, 75.64–79.43) protective immunity.^94^ Smaller studies from Italy, Spain and Luxembourg found similar results.^68^^,^^77^^,^^80^

Seven studies reported findings near the HIT (93–95%).^43^^,^^50^^,^  ^53^^,^^54^^,^^78^^,^^85^^,^^97^ In a study of 678 refugees in Germany, 92.6% had serological immunity reaching HIT levels [seronegativity: 7.4% (95% CI 5.5–9.6)].^53^ Additionally, a study found that 92.2% of 243 HIV-positive immigrants in Spain had protective immunity.^85^ However, two further studies demonstrated levels of protective immunity below the HIT: 89.9% (CI 87.3–92.4%) of 552 refugees in Germany^54^ and 88% (range: 83–93%) among 622 asylum seekers in the Netherlands, with the lowest protection levels in those under 25 years of age.^78^ Three studies found immunity above the HIT: a Swiss study in 1012 Latin American immigrants with 98.6% having immunity to measles,^97^ a Spanish study in 1374 immigrants with 96.5% having immunity to measles^84^ and a Greek study in a small sample of immigrants (*n =* 40) with 97.5% having immunity to measles.^66^

Four studies compared migrant immunity with that of the host population, and findings were mixed. Two studies found migrants to have significantly better vaccination coverage: one reported an adjusted hazard ratio of 1.12 (95% CI 1.03–1.22) for immunization uptake of refugee children compared with Danish-born children,^45^ and one found that measles seropositivity was significantly associated with migrant status [odds ratio (OR) 0.5 (95% CI 0.27–0.9)].^40^ While two studies found migrants to have significantly lower seropositivity: one an OR of 1.89 (95% CI 1.40–2.56) of being seronegative,^60^ and one reported an OR of 3.03 (95% CI 2.06–4.45) in migrants for being unvaccinated compared with the host population.^58^

**Figure 2:**
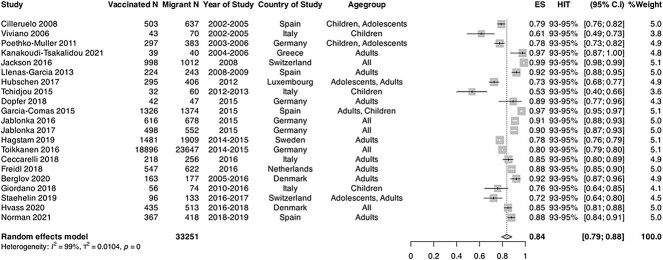
Forest plot of pooled immunity coverage for measles in migrant populations in EU/EEA countries. Pooled coverage/effective size (ES), *N = *number of migrants, V1 = number of migrants considered to be immune or vaccinated

Twenty-one studies that reported on serology/laboratory confirmed immune status immune status for measles were included in the meta-analyses.^43^^,^^44^^,^^50^^,^^53^^,^^54^^,^^59^^,^^63^^,^^66^^,^^68–70^^,^^73^^,^^77^^,^^78^^,^^80^^,^^84^^,^^85^^,^^87^^,^^94^^,^^95^^,^^97^ Pooled immunity coverage in the laboratory studies was 83.7% (95% CI: 79.9–88.2, *I*^2^ = 99%) (Figure 2), which is considerably below the HIT (93–95%). The range of measles seropositivity across studies included in the meta-analysis was 53.33–98.62%, indicating moderate heterogeneity. When carrying out subgroup analyses comparing serology data in children and adults, pooled seropositivity was 76.0% (*n =* 7; 95% CI: 68.8–83.4, *I*^2^ = 91%) in children compared with 88.7% (*n =* 11; 95% CI: 85.7–91.7, *I*^2^ = 93%) in adults. Sensitivity analyses in high-quality studies yielded pooled results compatible with the main analysis (88.13% [95% CI, 81.5–94.8], *I*^2^ = 99%).

### Mumps immunity

Seventeen studies (eight serology and nine vaccination history) reported on immunity to mumps.^40^^,^^45^^,^^46^^,^^48^^,^^53^^,^^56^^,^^57^^,^^60^^,^^65^^,^^69^^,^^73^^,^^77^^,^^78^^,^^80^^,^  ^83^^,^^85^^,^^87^ Mumps HIT is calculated at 88–93%, and we found that migrant populations in 11 studies had immunity status below the HIT levels, predominantly studies involving internationally adopted and refugee children. A study from Luxembourg found only 229 (56%) of 406 adolescent and adult migrants were considered to be immune, with significant variation by country of origin: 45.9% of migrants from the Balkans were considered not immune and 21.1% of African migrants.^77^ In Italy, a study found that only around half of the included internationally adopted children were not immune against mumps.^69^ Among 637 internationally adopted children in Spain, immunity coverage for mumps was low, with only 30% considered adequately vaccinated for mumps.^80^ The authors noted that the most frequent country of origin was China (46%), where administration of monovalent vaccines was common.^80^^,^^101^ Higher levels of protective immunity were found in a Dutch study, in which 91% (range: 80–97%) of 56 Eritrean and 92% (range: 83–97%) of 75 Afghani migrants were seropositive^78^ and a German study of 678 migrants of all ages reporting only 10.2% (95% CI 8.0–12.5%) were non-immune.^53^

Among the studies comparing immune status between migrants and host populations, a Danish registry study compared the intake of MMR doses administered at 15 months and 12 years between refugee children and adolescents to the host population. The study found that refugee children were slightly more likely to have had their first scheduled MMR vaccine (AHR 1.12 [95% CI 1.03–1.22]) administered at 15 months, though there was no difference for the second dose (HR 0.99 [95%CI 0.96–1.01]) administered later.^45^ A clear difference was found in an Austrian study, which showed that among 713 HIV-positive adults, migrants were significantly less likely to be seropositive for mumps (OR 0.57 [95% CI 0.4–0.8]).^40^

**Figure 3:**
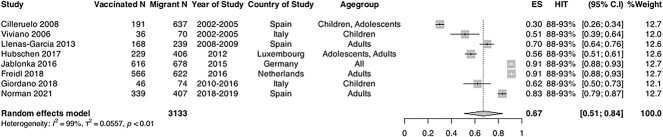
Forest plot of pooled immunity coverage for mumps in migrant populations in EU/EEA countries. Pooled coverage/effective size (ES), *N =* number of migrants, V1 = number of migrants considered to be immune or vaccinated

Eight studies that reported on serology/laboratory confirmed immune status immune status for mumps among migrants and were included in the meta-analysis.^53^^,^^69^^,^^73^^,^^77^^,^^78^^,^^80^^,^^85^^,^^87^ The estimated pooled immunity coverage was 67.1% (95% CI: 50.6–83.6; *I*^2^ = 99%) (Figure 3), suggesting that migrants are not sufficiently protected and that this group remains far below the population HIT. The range of mumps immunity coverage across studies included in the meta-analysis was 30.0–91.00%, indicating substantial heterogeneity. Pooled coverage was estimated to be lower in children compared with adults (*n =* 3, 47.4% [95% CI:26.6–69.1], I^2^ = 95% vs *n =* 3 81.8% [95% CI: 71.5–92.1], I^2^ = 96%), though CIs were wide. In sensitivity analyses, pooled coverage was higher (84.7% [95% CI: 76.8–92.7], I^2^ = 96%) in high quality studies.

### Rubella immunity

We identified 39 studies (22 serology and 17 vaccination history) on immunity to rubella,^40^^,^^43^^,^^45^^,^^46^^,^^48^^,^^50^^,^^53^^,^^54^^,^^56^^,^^57^^,^^60^^,^^63^^,^^65^^,^^67^^,^^69^^,^^71–74^^,^  ^77–82^^,^^84–96^^,^^99^ which predominantly focused on pregnant women (14 out of 39 studies).

In 14 studies, levels of protection among migrants fell below the estimated 83–94% HIT.^45^^,^^48^^,^^50^^,^^57^^,^^60^^,^^65^^,^^69^^,^^71–74^^,^^80^^,^^86^^,^^95^ In one Italian questionnaire-based study, rubella immunization rates were 36% among migrant women of childbearing age, compared with 60.2% among women born in Italy. In addition, 56.8% of immigrant women compared with 35.3% of Italian women did not know their rubella immunization status.^71^ A study of adopted children found a similar proportion (38%) had protective immunity.^80^ Slightly higher rates of protective immunity were reported among foreign-born pregnant women in a Spanish study (61.6%),^86^ and foreign-born children in a German study (79.6%; 95% CI 75.8–82.9), in which foreign-born children were found to be significantly more likely to be seronegative (OR: 2.19 [95% CI 1.71–2.82]).^60^ Especially low coverage was identified in recent migrants and those from Asia and Sub-Saharan Africa.^71^^,^^80^^,^^89^ A cohort study of children in Denmark also reported on immunization uptake, with 72% of refugee children receiving child health examinations and immunizations compared with 76% of Danish-born children.^45^

In a study with refugees in Germany, seronegativity for rubella was found to be only 2.2% (95% CI 1.2–3.4).^53^ In a second study with asylum seekers arriving in Germany, seroprevalence was found to be 85.1% (95% CI: 84.7–85.6) although males [87.3% (95% CI: 86.8–87.8] were significantly (*P* < 0.001) more protected than females [80.0% (95% CI: 78.9–81.1)]; and adults over 29 had higher immunity.^63^

Twenty-two studies reported immunity within or above the HIT estimated range,^43^^,^^46^^,^^53^^,^^54^^,^^56^^,^^67^^,^^77–79^^,^^81^^,^^82^^,^^84^^,^^85^^,^^87^^,^^88^^,^^90–94^^,^^96^^,^^99^ ranging from 86.2% to 95.8%. The majority of these studies focused on pregnant women, most of whom were receiving antenatal care, which may have included an MMR vaccination. Only three studies among pregnant women reported immunity below the HIT estimated range.^50^^,^^72^^,^^86^ Several studies found that host populations had higher levels of protection against rubella than migrants,^67^^,^^81^^,^^82^^,^^88^^,^^92^^,^^93^^,^^99^ particularly when compared with younger migrants and those from Asia and Africa.^77^^,^^81^^,^^99^

**Figure 4:**
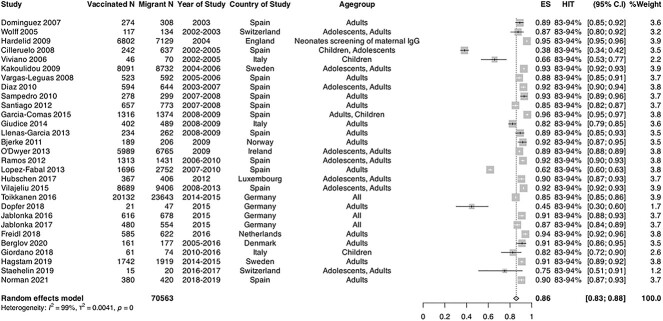
Forest plot of pooled immunity coverage for rubella in migrant populations in EU/EEA countries. Pooled coverage/effective size (ES), *N =* number of migrants, V1 = number of migrants considered to be immune or vaccinated

Twenty-nine studies that reported on serology/laboratory-confirmed immune status immune status for rubella were included in the meta-analysis^43^^,^^50^^,^^53^^,^^54^^,^^63^^,^^67^^,^^69^^,^^71^^,^^73^^,^^74^^,^^77–82^^,^^84–88^^,^ ^90–96^^,^^99^ with migrants estimated to have a pooled coverage of 85.6% (95% CI = 83.1–88.1%; *I*^2^ = 99%) (Figure 4). The range of rubella immunity coverage across studies included in the meta-analysis was 38.0–95.8%, indicating substantial heterogeneity. In these serology studies, pooled coverage was estimated to be lower in children compared with adults (69.6% [95% CI: 38.3–100.0], *I*^2^ = 99% vs 86.3% [95% CI: 82.2–90.3], *I*^2^ = 99%). In sensitivity analyses, pooled coverage was estimated to be 84.7% [(95% CI: 77.3–92.0), *I*^2^ = 99%] in high-quality studies.

### Diphtheria immunity

Fifteen studies reported on immunity to diphtheria,^41^^,^^42^^,^^46–49^^,^  ^57^^,^^64^^,^^65^^,^^75–78^^,^^80^^,^^98^ none of which reported levels of protection in migrants surpassing the HIT of 83–86%. Three seroprevalence studies including 1 of 250 Sub-Saharan Africans in France, 637 internationally adopted children in Spain and 620 asylum seekers in a Dutch study found rates of seropositivity to diphtheria at 69% (95% CI: 63–75%), 76% (95% CI: 72–79%)^80^ and 82% (95% CI: 65–88%)^78^ respectively. In contrast, a study from 2003 involving 1128 Afghani, Iraqi, Kurdish, Turkish and Kosovan refugees in southern Italy found only 54.8% had protective antibody levels, with the lowest proportion of immune individuals among Kurdish children from Turkey.^76^ A German study found that only 35.8% of 461 children and adolescent migrants aligned with the national vaccination schedule.^57^ Another German study by Hübschen *et al.* reported that of 406 newly arrived migrants arriving in Luxembourg, only 27% had adequate protection against diphtheria^77^.

We found only one study reporting a statistically significant difference between migrant and host populations, in which only 12.9% of North African-born migrants had been vaccinated compared with 37.8% of the French-born population (OR 0.43 [95% CI 0.17–1.09]).^47^

**Figure 5:**
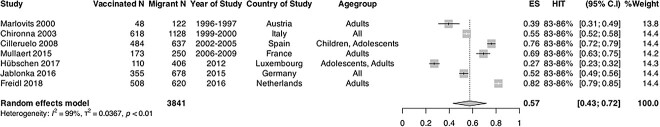
Forest plot of pooled immunity coverage for diphtheria in migrant populations in EU/EEA countries. Pooled coverage/effective size (ES), *N =* number of migrants, V1 = number of migrants considered to be immune or vaccinated

Seven studies reported on serology/laboratory-confirmed immune status for diphtheria and were included in the meta-analysis.^42^^,^^49^^,^^64^^,^^76–78^^,^^80^ Migrants were estimated to have a pooled immunity coverage of 57.4% (95% CI: 43.1–71.7; *I*^2^ = 99.6%), which is considerably below the HIT for diphtheria (Figure 5). The range of diphtheria immunity coverage across studies included in the meta-analysis was 27.1–82.0%, indicating substantial heterogeneity. Pooled coverage was estimated to be higher in children compared with adults (*n =* 3, 76.0% [95% CI: 72.5–79.3] vs *n =* 3 63.9% [95% CI: 42.9–84.8]), though confidence intervals were wide. In sensitivity analyses, pooled coverage was estimated to be 55.7% (95% CI: 31.5–80.0) in high-quality studies.

## Discussion

This systematic review and meta-analysis begin to address key gaps in the evidence base on the immune status of migrants in Europe. We found that pooled immunity coverage among migrant populations was below the recommended HIT for diphtheria (57.4% [95% CI: 43.1–71.7%] vs HIT 83–86%), measles (83.7% [95% CI: 79.2–88.21] vs HIT 93–95%) and mumps (67.1% [95% CI: 50.6–83.6] vs HIT 88–93%) and midway for rubella of 85.6% (95% [CI: 83.1–88.1%] vs HIT 83–94%), suggesting that migrants currently represent an under-immunized group who should be better engaged in catch-up vaccination initiatives on arrival for routine immunizations.

Our findings suggest that migrants represent a high-priority population for catch-up vaccination on or after arrival to ensure EU/EEA countries move towards elimination targets and to avoid outbreaks and cases of VPDs in these communities. Our findings align with other recent studies in specific groups of migrants showing under-immunization. For example, in a large recent analysis of refugees coming to the UK via a government resettlement programme, only 11% were fully aligned with the UK schedule for polio, 34% for measles and 5% for diphtheria and tetanus, with adults more likely than children to be under-immunized.^76^ One systematic review reported the odds of vaccination coverage among migrants were lower compared with non-migrants (summary OR 0.50; 95% CI 0.37–0.66; *I*^2^ 99.9%), calling on public health prevention programmes to prioritize vaccine equity.^102^ One European study identified 23 significant determinants of under-immunization in migrants in Europe (*P* < 0·05), including African origin, recent migration and being a refugee or asylum seeker.^103^ These data suggest that more research is needed to elucidate which particular nationality groups are most at risk for under-immunization and for which VPDs, to support better planning for arriving migrants and facilitate more targeted catch-up vaccination campaigns. Emphasis is also needed on newer vaccines such as HPV that are not widely available in the countries of origin of many migrant groups, to align them with European schedules and ensure vaccine equity. This must go hand in hand with increased engagement by front-line healthcare professionals to ascertain vaccination history and to deliver required vaccines in these populations, with appropriate training and resources. It is also important to note that although immunity among migrants was found to be low, this finding occurs in a context of sub-optimal and declining vaccine coverage in the general population. In addition, under-immunized groups are not limited to migrants, with renewed focus now being placed on several under-immunized groups in the European context. While it is important to recognize migrants as an under-immunized group, closing the immunity gap among migrants should be part of a broader inequity strategy that seeks to address inequities in vaccination among all these groups.

Against the background of increasing VPD outbreaks leading to avoidable deaths and disability, there have been calls to review the current approach to vaccination of migrant populations in Europe.^104^ One pan-European study of 32 EU/EEA vaccination experts reported that guidance to front-line healthcare staff on vaccination approaches was not migrant-specific and rarely applied in practice. Low levels of catch-up vaccination were reported in adult migrants specifically, with only 13 countries offering MMR and 10 countries charging fees to migrants.^23^ In 30 countries, child migrants without evidence of previous vaccination were re-vaccinated according to the national schedule. In a policy analysis of migrant vaccination in 32 EU/EEA countries,^105^ 10 (31.3%) countries’ policies focused on priority vaccinations, and heterogeneity was noted in vaccines recommended to adults, adolescents and children. Specific WHO guidance for catch-up vaccination is available, but evidence suggests it is poorly implemented in practice.^106^ In Europe, the ECDC has recently published guidance on catch-up vaccination for adult, adolescent and child migrants arriving to European countries.^107^^,^^108^ This guidance requests that healthcare providers consider revaccinating child, adolescent and adult migrants with uncertain vaccination status or no recorded history of vaccination for measles, mumps, rubella and diphtheria, tetanus and polio. Effective implementation of these guidelines will require training, supporting and resourcing healthcare staff in delivering life-course vaccination in migrant groups among those with uncertain or incomplete vaccination status, as well as meaningful engagement with migrant communities through culturally appropriate vaccination support materials to promote demand and uptake and address hesitancy and barriers to vaccine services.^9^^,^^109^

This systematic review represents an attempt to systematically and comprehensively examine the immune status of migrants in Europe. However, there are limitations that need to be noted when considering the results. The key limitation of this study was the substantial heterogeneity in immunity coverage estimates across studies. Although the meta-analyses brought forward very strong pooled results for measles and rubella, the pooled results for mumps and diphtheria may be less representative given the limited data available in a small number of studies. In general, across all included studies there was significant heterogeneity in the data (*I*^2^ statistic was over 90% for all meta-analyses) and the range across studies varied widely. The high heterogeneity was likely attributed to variations in study methods, migrant populations included, country of origin, migration route, socioeconomic status, reason for migration and testing method for vaccination/immune status, which must be taken into consideration when interpreting the results, noting that the quality of included studies was generally high. This is a common problem in the migrant health field because of historically poor data collection and lack of large-scale studies and trials. For instance, 23 studies included only sub-populations (13 pregnant women and one HIV patient) which narrows the external validity of the results. In particular, the immune status for rubella may be overestimated at 85.6%, as 13 out of 29 studies (45%) included in the meta-analysis only involved pregnant women, which is a group that is likely to be targeted during routine antenatal care. It is possible that in the general population, rubella immunity is much lower. In addition, there may be inherent heterogeneity of serological results and their interpretation. We therefore urge readers to be cautious when interpreting the pooled immunity coverage estimates from the meta-analyses due to inherent heterogeneity considerations.

Going forward, it will be important to conduct more research that ascertains which specific migrant groups are most at risk, linking data to the quality of vaccination systems in their countries of origin; however, data were insufficient in these studies to make any broad conclusions about specific nationality groups. Furthermore, the diversity in political environments and geography of host countries strongly influences migration patterns as well as health systems, which makes it difficult to draw general conclusions. Some studies were less transparent or comprehensive in reporting their findings, resulting in not all data being included in the separate meta-analyses.

Data on the immune status of migrants on key infectious diseases is critical to inform evidence-based catch-up vaccination policies in EU/EEA countries. A migrant’s ability to get catch-up vaccinations and other preventative healthcare services is often restricted by disparities in access to mainstream health services and inconsistencies in delivery of care.^3^^,^^12^^,^^110^ The COVID-19 pandemic highlighted multiple access and uptake barriers in these populations that will need to be better considered in order to improve coverage in these groups.^9^^,^^10^^,^^103^ Many studies describe well-known barriers in the provision of healthcare services for migrants, including language barriers, cultural and communication barriers and low levels of literacy, combined with a lack of experience and knowledge around a host country’s health system among migrant patients.^111–113^ Our data suggest that catch-up vaccination initiatives may need to be targeted at specific migrant groups, nationalities or migrants from specific regions of the world, who are at high risk of under-immunization. Improving vaccine coverage will require working with affected populations to co-design vaccination strategies to ensure that they are tailored to the diverse needs of migrant communities and build trust and confidence in vaccines through continuous, inclusive engagement.^114^ In addition, focus should be placed on developing new migrant-inclusive models of vaccine service delivery. These should draw on innovations in service delivery models seen during the COVID-19 pandemic to increase uptake, including outreach; out-of-hours services; and delivery of vaccines in faith-based venues, community-based venues and trusted locations, alongside specific vaccination campaigns to provide culturally and linguistically tailored materials around the benefits of routine immunizations across the life course.

## Supplementary Material

NewSupplementaryFileCLEANJTravMed2023_taae033(1)
