# Supplementary material for: The immune status of migrant populations in Europe and implications for vaccine-preventable disease control: a systematic review and meta-analysis
Source: J Travel Med. 2024 Feb 29;31(6):taae033. doi: 10.1093/jtm/taae033 (PMC11790012; doi:10.1093/jtm/taae033)
Supplement: NewSupplementaryFileCLEANJTravMed2023_taae033(1) [file newsupplementaryfilecleanjtravmed2023_taae033(1).docx]

**Supplementary information**

**The immune status of migrant populations in Europe and implications for vaccine-preventable disease control: a systematic review and meta-analysis**

Zeinab Cherri*, Karen Lau*, Laura B Nellums*, Jan Himmels*, Anna Deal, Emma McGuire, Sandra Mounier-Jack, Marie Norredam, Alison Crawshaw, Jessica Carter, Farah Seedat, Nuria Sanchez-Clemente, Oumnia Bouaddi, Jon S Friedland, Michael Edelstein, Sally Hargreaves

*Joint first authors

Correspondence to: Dr Sally Hargreaves [s.hargreaves@sgul.ac.uk](mailto:s.hargreaves@sgul.ac.uk)

***Table S1. Characteristics of all included studies in the systematic review and meta-analyses***

| Author/Ref | | Year of Study | Country of Study | Disease reported on | Migrant type | Migrant nationality/origin | Age group | Assessment of Immune Status | Setting | Included in meta-analysis? | Quality Assessment Score^a^ |
| --- | --- | --- | --- | --- | --- | --- | --- | --- | --- | --- | --- |
| Berglov 2020 | | 2005-2016 | Denmark | Measles, Rubella | Women with Chronic Hepatitis B | Worldwide | Adults | Serology | Primary care-hospital | Yes | 6 |
| Bjerke 2011 | | 2009 | Norway | Rubella | Pregnant women | Pakistan | Adults | Serology | Primary care-  hospital | Yes | 4 |
| Bonito 2021 | | 2019-2020 | Italy | Diphtheria | Pregnant women | Worldwide | Adults | Register Data | Primary care-  hospital | No | 6 |
| Ceccarelli 2018 | | 2016 | Italy | Measles | Asylum seekers | Eritrea, Mali, Gambia, Senegal, Nigeria, Pakistan, Bangladesh | Adults | Serology | Asylum centre | Yes | 5 |
| Chironna 2003 | | 1999-2000 | Italy | Diphtheria | Refugees | Afghanistan, Iraq, Kurdistan, Turkey, Kosovo | All | Serology | Refugee camp | Yes | 7 |
| Cilleruelo 2008 | | 2002-2005 | Spain | MMR, DTP | Internationally  adopted children | Worldwide (46% from China) | Children,  Adolescents | Serology | Primary care –  hospital | Yes | 6 |
| Díaz 2010 | | 2003-2007 | Spain | Rubella | Women of  childbearing age | Worldwide | Adolescents,  Adults | Serology | Primary care –  hospital | Yes | 5 |
| Dominguez 2007 | | 2003 | Spain | Rubella | Pregnant Women | Worldwide | Adults | Serology | Primary care-  hospital | Yes | 5 |
| Dopfer 2018 | | 2015 | Germany | Measles, Rubella | Refugees, Pregnant Women | Worldwide | Adults | Serology | Refugee centre | Yes | 5 |
| Fabiani 2017 | | 2009-2014 | Italy | Rubella | Women of  childbearing age | Worldwide | Adults | Register Data | Not stated | No | 6 |
| Facciola 2019 | | 2018 | Italy | Rubella | Pregnant women | Worldwide | Adults | Self-reported  questionnaire | Primary care-hospital | No | 3 |
| Freidl 2018 | | 2016 | Netherlands | MMR, DTP | Asylum seekers | Syria, Iran, Iraq,  Afghanistan, Eritrea, Ethiopia | Adults | Serology | Asylum centre | Yes | 6 |
| Fuhrer 2016 | | 2015 | Germany | Measles | Asylum seekers | Worldwide | Adults, Children | Self-reported  questionnaire | Asylum centre | No | 3 |
| García 2007 | | 2005 | Spain | Mumps | Not stated | Not Stated | All | Register Data,  Serology | Not stated | No | 5 |
| Garcia-Comas 2015 | | 2008-2009 | Spain | Measles, Rubella | Not stated | Not Stated | Adults, Children | Serology | Primary care | Yes | 5 |
| Gautret 2010 | | 2006 | France | Diphtheria | Pilgrims to Mecca | North Africa (Algeria, Morocco, Tunisia) | Adults | Self-reported, vaccination certificate | Primary care-hospital | No | 6 |
| Giordano 2018 | | 2010-2016 | Italy | MMR | Internationally  adopted children | Worldwide | Children | Clinical records, Serology | University hospital | Yes | 5 |
| Giudice 2014 | | 2008-2009 | Italy | Rubella | Women of childbearing age | Sri Lanka, Philippines, India, Romania, Poland, Kosovo, Ukraine, Russian Federation, Morocco, Tunisia, Libya, Algeria | Adults | Serology, self-reported questionnaire | University hospital | Yes | 5 |
| Grabmeier-Pfisterhammer  2015 | | 2012-2013 | Austria | DTP | HIV-positive | Worldwide | Adults | Serology | Primary care -  hospital | No | 7 |
| Grabmeier-Pfisterhammer  2014 | | 2012-2013 | Austria | MMR | HIV-positive | Worldwide | Adults | Serology | Primary care -  hospital | No | 5 |
| Hagstam 2019 | | 2014-2015 | Sweden | Measles, Rubella | Asylum seekers, Antenatal screening | Worldwide | Adults | Serology | University hospital | Yes | 6 |
| Hardelid 2009 | | 2004 | UK | Rubella | Pregnant women | Worldwide | Neonates  (maternal IgG) | Serology | Primary care | Yes | 5 |
| Heuvel 2020 | | 2018 | Germany | Measles | Asylum seekers | Worldwide | All | Register Data | Asylum centre | No | 4 |
| Hübschen 2017 | | 2012 | Luxembourg | MMR, DTP | Refugees/Asylum  seekers | Worldwide | Adolescents,  Adults | Serology | On arrival | Yes | 5 |
| Hvass 2020 | | 2016-2018 | Denmark | Measles | Refugees | Syria, Iran, Iraq, Afghanistan, Pakistan, Eretria, Ethiopia, Somalia, Lebanon, Pakistan, Russia, Palestine, Morocco, Jordan | All | Serology | Asylum centre | Yes | 5 |
| Jablonka 2016 | | 2015 | Germany | MMR | Refugees | Worldwide | All | Serology | Asylum centre | Yes | 6 |
| Jablonka 2016 | | 2015 | Germany | Diphtheria | Refugees | Worldwide | All | Serology | Asylum centre | Yes | 7 |
| Jablonka 2017 | | 2015 | Germany | Measles, Rubella | Refugees | Worldwide | All | Serology | Asylum centre –  on admittance | Yes | 5 |
| Jackson 2016 | | 2008 | Switzerland | Measles | Various | Latin America | All | Serology | Primary health  care | Yes | 6 |
| Jones 2016 | | 2016 | France | Measles | Refugees | Worldwide | All | Clinical records | Calais Refugee  camp | No | ND |
| Kakoulidou 2009 | | 2004-2006 | Sweden | Rubella | Pregnant women | Worldwide | Adolescents,  Adults | Serology | Primary care-  hospital | Yes | 5 |
| Kanakoudi-Tsakalidou 2021 | | 2015-2019 | Greece | Measles | Mothers with acute respiratory or gastrointestinal infections not affecting the general condition Urinary tract infection and their full-term infants | Albania, Russia, Georgia, Italy, Germany, Poland, Bulgaria, Rumania, Ukraine, and Armenia. | Adults | Self-reported  questionnaire, Serology | University hospital | Yes | 6 |
| Kühne 2016 | | 2004-2014 | Germany | Measles | Asylum seekers | Outbreak - Check all | All | Register Data | Primary care-  hospital | No | ND |
| Llenas-García 2013 | | 2008-2009 | Spain | MMR | HIV-positive | Worldwide | Adults | Serology | Primary care-  HIV unit | Yes | 6 |
| López-Fabal 2013 | | 2007-2010 | Spain | Rubella | Pregnant women | Worldwide | Adults | Serology | Primary care-  hospital | Yes | 3 |
| Loucas 2018 | | 2015-2016 | Germany | MMR, DTP | Refugees | Worldwide (predominantly Syria and Afghanistan) | Children,  Adolescents | Medical Records | Refugee centre | No | 5 |
| Mansor-Lefebvre 2020 | | 2013 | France | MMR, DTP | Homesless children, Asylum seekers | Worldwide | Children | Self-reported questionnaire, vaccination card | Asylum centre | No | 7 |
| Marlovits 2000 | | 1996-1997 | Austria | Diphtheria | Injured Adults | Worldwide | Adults | Serology | Primary care-  hospital | Yes | 7 |
| Mellou 2019 | | 2017-2018 | Greece | MMR, DTP | Refugees | Worldwide | Children,  Adolescents | WHO booklet, documentation | Mainland camps | No | 3 |
| Meinel 2016 | | 2015 | Switzerland, Germany | Diphtheria | Refugees | Eritrea, Ethiopia, Somalia, Syria | Adolescents, Adults | PCRᵇ | Primary care-  hospital | No | 5 |
| Moller 2016 | | 1993-2010 | Denmark | MMR | Refugees | Worldwide | Children,  Adolescents | Register Data | Not stated | No | 8 |
| Mullaert 2015 | | 2006-2009 | France | Diphtheria | HIV-positive | Sub-Saharan African (Central and West Africa) | Adults | Serology | Primary care-  hospital | Yes | 7 |
| Nakken 2018 | | 2015 | Denmark | MMR, DTP | Asylum seekers | Syria, Afghanistan, Russia, Stateless Palestinians, Iran, Iraq, Eritrea, Somalia | Children,  Adolescents | Clinical records | Red Cross Centers | No | 5 |
| Nehring 2021 | | 2014 | Germany | MMR | Refugees | Syrian | Children,  Adolescents | Self-reported questionnaire | Reception camp | No | 5 |
| Norman 2021 | | 2018-2019 | Spain | MMR | Various | Worldwide | Adults | Serology | Specialized unit | Yes | 4 |
| O'Dwyer 2013 | | 2009 | Ireland | Rubella | Pregnant women | Non-EU | Adolescents, Adults | Serology | Not stated | Yes | ND |
| Perry 2020 | | 2014-2018 | UK | Measles | Asylum seekers | Not Stated | Children, Adolescents | Register Data | Asylum centre | No | 5 |
| Poethko-Müller 2009 | | 2003-2006 | Germany | Measles | Not stated | Worldwide | Adolescents | Self-reported  questionnaire | Not stated | No | 5 |
| Poethko-Müller 2011 | | 2003-2006 | Germany | Measles | Not stated | Worldwide | Children,  Adolescents | Serology | Not stated | Yes | 6 |
| Poethko-Müller 2012 | | 2003-2006 | Germany | MMR | Not stated | Worldwide | Children,  Adolescents | Serology | Not stated | No | 5 |
| Ramos 2012 | | 2006-2010 | Spain | Rubella | Pregnant Women | Worldwide | Adolescents, Adults | Serology | Primary care-  hospital | Yes | 6 |
| Roggendorf 2012 | | 2010 | Germany | Measles | Not stated | Bulgarian (index  cases) | All | PCR^b^ | Primary care- Community health centre | No | ND |
| Sampedro 2010 | | 2007-2008 | Spain | Rubella | Pregnant women | Worldwide | Adults | Serology | Primary care | Yes | 4 |
| Santiago 2012 | | 2007-2008 | Spain | Rubella | Pregnant women | Worldwide | Adults | Serology | Primary care-  hospital | Yes | 5 |
| Staehelin 2019 | | 2016-2017 | Switzerland | Measles, Rubella | Women of childbearing age | Eritrea | Adolescents, Adults | Serology | Asylum centre | Yes | 5 |
| Takla 2012 | | 2010 | Germany | Measles | Asylum seekers | Worldwide | All | Serology | Asylum centre | No | ND |
| Tchidjou 2015 | | 2012-2013 | Italy | Measles | Internationally  adopted children | Worldwide | Children | Serology | Primary care -  hospital | Yes | 5 |
| Toikkanen 2016 | | 2014-2015 | Germany | Measles, Rubella | Asylum seekers | Worldwide | All | Serology | On arrival | Yes | 5 |
| Vargas-Leguas 2008 | | 2005-2006 | Spain | Rubella | Pregnant women | Worldwide | Adults | Serology | Not stated | Yes | 6 |
| Vilajeliu 2015 | | 2008-2013 | Spain | Rubella | Pregnant women | Worldwide | Adolescents, Adults | Serology | Primary care -  hospital | Yes | 5 |
| Viviano 2006 | | 2002-2005 | Italy | MMR | Internationally  adopted children | Ukraine, Russia, Poland, Rumania, Bulgaria, Hungary, India | Children | Serology | Adoption centre | Yes | 5 |
| Wolff 2005 | | 2002-2003 | Switzerland | Rubella | Pregnant women | Worldwide | Adolescents, Adults | Serology | Primary care | Yes | 6 |
|  | ^a^ Quality assessment was performed using the Joanna Briggs Institute (JBI) critical appraisal tool for cross-sectional and cohort studies. A total of eight points could be allocated to each study, with scores of 6 to 8 considered high quality.  ^b^ Outbreak confirmed by PCR. | | | | | | | | | | |

***Supplementary information: Examples of search terms and strategy used in database search***

**Web of Science:**

**(**

**(**Measles* or MMR* or MCV* or Priorix* or Tresivac* or Trimovax* or Priorix Tetra* or ProQuad* or Meruvax* or Mumpsvax* or Attenuvax* or Mumps* or Rubella* or Diphtheria* or Diphtheritic* or DPT* or DTP* or DTwP* or DTaP* or Tdap* or DKTP* or Corynebacterium diphtheriae*

**)**

**AND**

(Migration* or immigration* or emigration* or migrant* or immigrant* or emigrant* or asylum* or refugee* or undocumented* or ((foreign* or irregular* or non-resident* or transient* or visa* or origin* or non-citizen* or citizenship) AND nationalit*)

**AND**

(Austria* or Belgi* or Bulgaria* or Cyprus or Cypriot or Czech* or Denmark or Danish or Estonia* or Finland or Finnish or France or French or German* or Gree* or Hungar* or Iceland* or Ireland or Irish or Italy or Italian or Latvia* or Liechtenstein or Lithuania* or Luxembourg* or Malta* or Netherlands or Dutch or Norway or Norwegian or Poland or Polish or Portug* or Romania* or Slovakia* or Slovenia* or Spain* or Spanish or Sweden or Swedish or Britain or England or British or English or Wales or Welsh or Scotland or Scottish or EU or EEA or Europe*)

)

**OVID:**

**(**Measles* or MMR* or MCV* or Priorix* or Tresivac* or Trimovax* or Priorix Tetra* or ProQuad* or Meruvax* or Mumpsvax* or Attenuvax* or Mumps* or Rubella* or Diphtheria* or Diphtheritic* or DPT* or DTP* or DTwP* or DTaP* or Tdap* or DKTP* or Corynebacterium diphtheriae***)**

**AND**

(Migration* or immigration* or emigration* or migrant* or immigrant* or emigrant* or asylum* or refugee* or undocumented* or ((foreign* or irregular* or non-resident* or transient* or visa* or origin* or non-citizen* or citizenship) AND nationalit*))

**AND**

(Austria* or Belgi* or Bulgaria* or Cyprus or Cypriot or Czech* or Denmark or Danish or Estonia* or Finland or Finnish or France or French or German* or Gree* or Hungar* or Iceland* or Ireland or Irish or Italy or Italian or Latvia* or Liechtenstein or Lithuania* or Luxembourg* or Malta* or Netherlands or Dutch or Norway or Norwegian or Poland or Polish or Portug* or Romania* or Slovakia* or Slovenia* or Spain* or Spanish or Sweden or Swedish or Britain or England or British or English or Wales or Welsh or Scotland or Scottish or EU or EEA or Europe*)
